# Arteriovenous fistula after temporomandibular joint arthroscopy treated with precipitating hydrophobic injectable liquid: case report and literature review

**DOI:** 10.1007/s10006-026-01553-5

**Published:** 2026-04-01

**Authors:** Alex Lederman, Bruno Reis, Ricardo Grillo, Fernando Melhem-Elias

**Affiliations:** 1 Department of Vascular Surgery, Universitary Hospital, University of São Paulo, São Paulo, Brazil; 2 Department of Oral and Maxillofacial Surgery, School of Dentistry, University of São Paulo, São Paulo, Brazil; 3 Faculdade de Odontologia, University of São Paulo, Av. Prof. Lineu Prestes, 2227. Cidade Universitária, São Paulo, 05508-000 SP Brazil

**Keywords:** Temporomandibular joint disorders, Arthroscopy, Arteriovenous fistula, Embolization, Therapeutic, Bleeding

## Abstract

**Introduction:**

Arteriovenous fistulas (AVFs) are abnormal connections between the arterial and venous systems, bypassing the capillary network and causing high-flow shunting. While AVFs can be congenital, spontaneous, or traumatic, trauma remains the most common cause. Although iatrogenic AVFs after temporomandibular joint (TMJ) procedures are rare, their recognition is crucial due to potential complications such as hemorrhage, thromboembolism, and high-output cardiac failure. This report presents a rare case of an AVF following TMJ arthroscopy and discusses its management using superselective arterial embolization with Precipitating Hydrophobic Injectable Liquid (PHIL).

**Case report:**

A 24-year-old man with TMJ internal derangement underwent TMJ arthroscopy. After the procedure, massive pulsatile bleeding occurred, leading to early suspension of surgery. Two years later, the patient presented with a pulsatile sound, diminished hearing, and right-sided pain. CT angiography revealed an AVF with three feeding branches and a high-flow fistula involving the superficial temporal artery. Selective angiography and subsequent superselective embolization using PHIL completely occluded the fistula. Follow-up angiography confirmed successful embolization, and the patient reported resolution of symptoms, remaining asymptomatic at 8 months.

**Conclusion:**

Although AVFs following TMJ arthroscopy are rare, early recognition and appropriate treatment are crucial. Superselective arterial embolization with PHIL represents an effective, minimally invasive treatment for TMJ-associated AVFs. This case highlights the importance of prompt intervention in managing vascular complications following TMJ arthroscopy and demonstrates PHIL’s potential as an embolic agent in high-flow fistulas.

**Clinical relevance:**

Superselective embolization with PHIL offers a precise, minimally invasive solution for high-flow AVFs post-TMJ arthroscopy, ensuring complete occlusion while minimizing complications. This case underscores PHIL’s efficacy as an advanced embolic agent for rare but critical vascular injuries, optimizing patient outcomes.

**Supplementary Information:**

The online version contains supplementary material available at 10.1007/s10006-026-01553-5.

## Introduction

Arteriovenous fistulas (AVFs) are abnormal communications between the arterial and venous systems, bypassing the capillary network and resulting in high-flow shunting [1]. While AVFs can be congenital, spontaneous, or traumatic, trauma remains the most common etiology [2]. The external carotid artery and its branches, particularly the superficial temporal artery, are frequently involved in post-traumatic AVFs, with documented cases occurring after blunt or penetrating head and neck injuries. However, iatrogenic AVFs following temporomandibular joint (TMJ) procedures are exceedingly rare [3].

TMJ arthroscopy is widely regarded as a minimally invasive and effective technique for managing internal derangement and inflammatory disorders of the TMJ. Despite its favorable safety profile, vascular complications such as pseudoaneurysms and AVFs have been reported, albeit infrequently [4]. Although only a limited number of case reports have documented AVFs following TMJ arthroscopy, their recognition is critical due to potential complications, including hemorrhage, thromboembolism, and high-output cardiac failure [5]. Imaging modalities such as digital subtraction angiography remain the gold standard for diagnosis, while noninvasive techniques like CT angiography and MR angiography provide valuable adjunctive assessment. Treatment options range from open surgical ligation to endovascular embolization, the latter being increasingly preferred due to its minimally invasive nature and high success rates [6].

In this report, we present a rare case of an AVF involving the superficial temporal artery following TMJ arthroscopy. We discuss its clinical presentation, diagnostic evaluation, and successful management with superselective arterial embolization, highlighting the importance of early recognition and intervention in optimizing patient outcomes.

## Case report

A 24-year-old man without comorbidities underwent right TMJ arthroscopy at an outside institution for management of internal derangement. According to the patient’s recollection, inflow and outflow ports were inserted based on the Holmlund-Hellsing line, and the joint space was irrigated with Ringer’s solution. Upon removal of the inflow port, massive pulsatile bleeding was noted. Due to difficulty controlling the hemorrhage, the procedure was suspended. Immediately postoperatively, the patient complained of a pulsatile sound. The surgeon advised that this was due to postoperative inflammation and expected spontaneous resolution, recommending observation rather than investigation. The operative duration, specific arthroscopy level, and whether vascular injury was suspected intraoperatively remain unknown, as the surgical report was unavailable for review.

The patient experienced these symptoms continuously throughout the following two years but did not seek further medical attention. This delay was multifactorial: relocation to a different city, financial constraints, and had reassurance by the initial surgeon that symptoms would likely resolve. Over time, the patient developed a palpable preauricular thrill and constant debilitating vertigo.

Two years postoperatively, the patient was referred to our service for management of a suspected vascular complication. At presentation, the patient reported a loud, right-sided constant pulsatile fremitus, diminished hearing, and persistent pain. The original medical records remained unobtainable despite multiple attempts, and the initial surgeon could not be contacted for additional technical details.

**Fig. 1:**
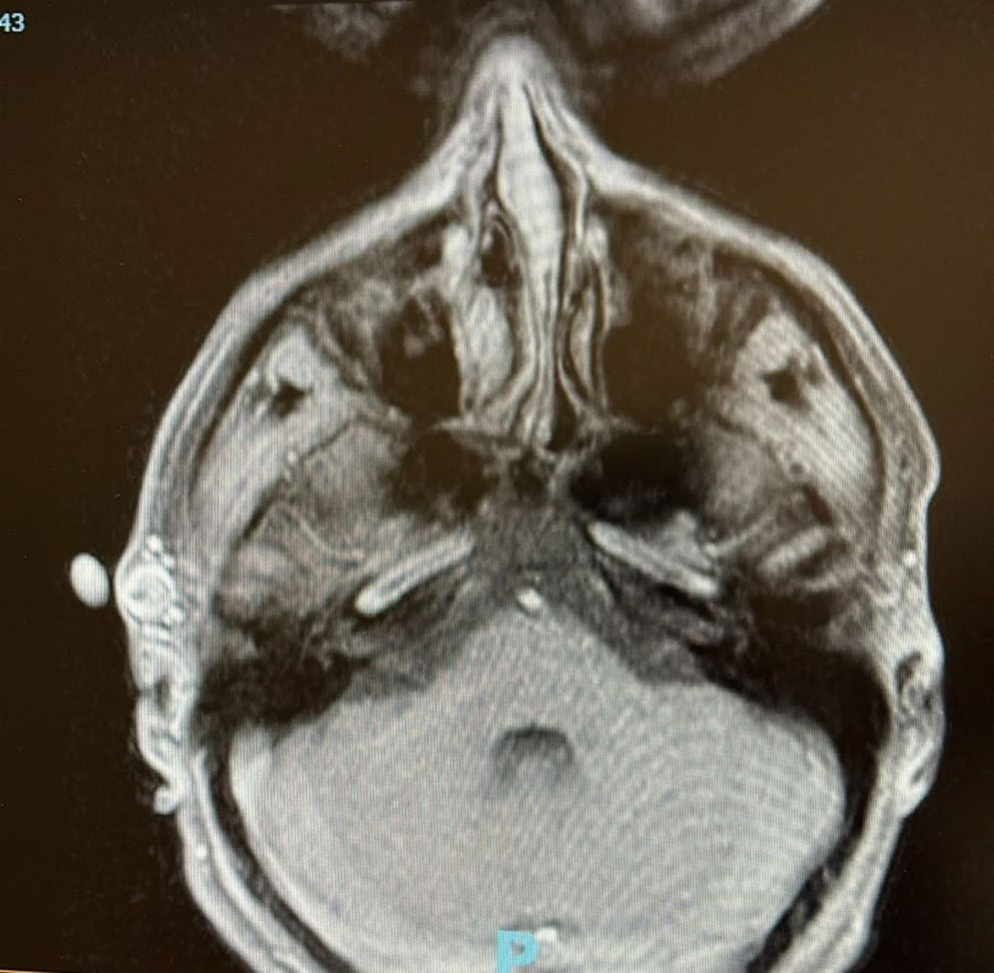
Post-embolization imaging outcome showing complete occlusion of the fistulous communication with preservation of surrounding arterial branches

After the initial CT angiography, an arteriovenous fistula (AVF) with three feeding branches was identified. No prior imaging studies had been performed during the two-year interval despite these persistent clinical complaints, as the patient had not sought care and no referring provider had ordered investigations. There was dilation of the face, with rapid venous filling and venous drainage following the injection via the arterial route. Through standard right-sided 5 F femoral access, a 5 F vertebral guide catheter was employed to conduct selective angiographic studies of the intracranial vasculature and both external carotid arteries. Injection into the ipsilateral external carotid artery revealed a high-flow, direct arteriovenous fistula in the superficial temporal region. No distinct anomalous anastomoses between the external and internal carotid arteries were observed, and no other contributions to the fistula were identified, except for the supply from the left-sided external carotid. Under systemic heparinization, a microcatheter (6 × 7 mm) was introduced coaxially over a 0.014 micro-guidewire, allowing for selective catheterization of the superficial temporal artery. Injections from the microcatheter at this site confirmed its accurate positioning at the fistula’s location. The AVF was embolized via the external carotid artery using two units of Precipitating Hydrophobic Injectable Liquid (PHIL) (Supplemental videos). After control angiography, retrograde flow continued to perpetuate the fistula through the parietal branches. A direct puncture was performed, and an additional unit of PHIL was used for embolization (Fig. 1). Follow-up angiography revealed complete obliteration of the fistula, with no reflux into the treated fistulous communication. Arterial sealing was achieved using an AngioSeal. The patient reported an immediate resolution of the pulsatile fremitus, vertigo, and hearing loss at the end of the procedure, confirmed by a follow-up angiogram. At an 8-month follow-up, the patient remained asymptomatic regarding the fistula. A repeat computed tomographic angiography at that time confirmed the complete resolution of the fistula.

## Discussion

Arthroscopic surgery of the temporomandibular joint (TMJ) is generally considered a safe and minimally invasive procedure [5]. However, despite its low complication rates, vascular injuries, including arteriovenous fistulas (AVFs) and false aneurysms, can arise as rare but significant complications [3, 7]. Due to its uncommon occurrence the literature on the topic is scarce. The occurrence of such vascular injuries is predominantly associated with trauma to the superficial temporal artery and vein during surgical instrumentation [2]. While most bleeding complications during arthroscopy are manageable through direct pressure or local measures, undetected arterial or venous trauma can lead to postoperative sequelae, such as the formation of AVFs or pseudoaneurysms, necessitating more aggressive intervention.

**Fig. 2:**
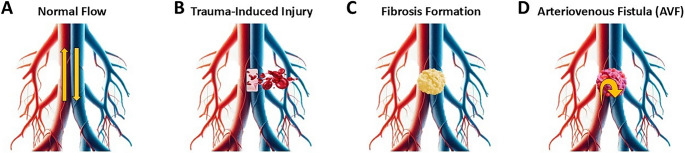
Pathophysiological progression from normal vascular anatomy to arteriovenous fistula following traumatic injury. (**A**) Blood flows from arteries to capillaries and then to veins, ensuring proper oxygen exchange. (**B**) Damage to adjacent arteries and veins disrupts normal flow, creating a risk of abnormal connections. (**C**) The body’s reparative response leads to fibrosis and endothelial proliferation, stabilizing the injury site. (**D**) An abnormal artery-vein connection bypasses capillaries, causing turbulent flow and vessel dilation

An AVF forms when an abnormal connection develops between an artery and a vein, bypassing the normal capillary network. This can occur due to trauma, such as surgical instrumentation or penetrating injury, which damages adjacent arterial and venous structures. In response to injury, the body initiates a reparative process involving fibrosis and endothelial proliferation, which can inadvertently stabilize the abnormal communication. The high-pressure arterial system forces blood directly into the low-pressure venous system, resulting in turbulent flow and increased shear stress on vessel walls. Over time, this altered hemodynamics can lead to vessel dilation, progressive enlargement of the fistula, and potential complications such as venous hypertension, steal phenomenon, or congestive symptoms (Fig. 2). The severity of these effects depends on the size and location of the AVF, as well as the relative pressure gradients between the connected vessels [4, 5, 8, 9]. Doppler ultrasound is a non-invasive diagnostic method that identifies the fistula, demonstrating continuous wave flow and systolic enhancement (typical of an AVF).

AVFs, in particular, pose a challenge due to their rapid hemodynamic changes. Clinically, this manifests as a pulsatile mass with a palpable thrill and an audible bruit [1, 4]. The mechanism behind AVF formation following TMJ arthroscopy is thought to involve either sharp or blunt trauma to the superficial temporal artery, potentially extending to the highly vascular pterygoid venous plexus. The resultant pressure differentials between the arterial and venous circulation can exacerbate vessel dilation and lesion progression.

Despite its rarity, the literature highlights cases where AVFs have developed after TMJ arthroscopy, reinforcing the need for heightened awareness and early detection [4, 7]. Arteriography remains the gold standard for diagnosis, allowing precise anatomical localization of the lesion and facilitating appropriate treatment planning. However, noninvasive imaging modalities such as CT angiography and MR angiography have gained traction as viable alternatives, offering detailed vascular assessment with reduced procedural morbidity [5].

Management of TMJ arthroscopy-associated AVFs typically involves either surgical or endovascular approaches [4, 5, 7, 9]. Historically, surgical ligation and excision of the AVF were the primary treatment modalities. However, these techniques carry inherent risks, including significant intraoperative hemorrhage and incomplete obliteration of the lesion due to collateral vascular contributions. More recently, superselective arterial embolization has emerged as the preferred treatment strategy due to its minimally invasive nature and reduced morbidity. Embolization effectively reduces blood flow to the AVF, thereby facilitating subsequent surgical resection if necessary [6]. Various embolic materials, including Gelfoam, polyvinyl alcohol particles, and metallic coils, have been employed with success.

While therapeutic embolization is highly effective, it is not devoid of complications. Risks include stroke, ischemia, neurological deficits due to unintended embolization, and foreign body reactions to embolic materials. Therefore, careful patient selection, meticulous technique, and real-time imaging guidance are paramount in optimizing outcomes. In cases where embolization alone is insufficient, combined approaches incorporating both embolization and surgical excision may be warranted to ensure complete resolution of the AVF.

A novel aspect of this report is the utilization of Precipitating Hydrophobic Injectable Liquid (PHIL) as an embolic agent for the management of TMJ arthroscopy-associated AVFs. PHIL is an innovative, non-adhesive, liquid embolic material composed of a copolymer dissolved in dimethyl sulfoxide (DMSO), which solidifies upon contact with blood, leading to controlled vessel occlusion [10]. Unlike particulate or coil-based embolization, PHIL provides a homogenous and durable embolization effect, reducing the risk of recanalization while allowing for precise deployment under fluoroscopic guidance.

Several liquid embolic agents are currently available for endovascular embolization, each with distinct characteristics, advantages, and limitations [11]. Cyanoacrylates (n-BCA glue), the earliest agents used for embolization, polymerize rapidly upon contact with blood and provide durable occlusion but carry a risk of catheter entrapment and require significant operator expertise due to their adhesive nature. Onyx, an ethylene-vinyl alcohol copolymer dissolved in DMSO, became the first broadly used non-adhesive agent and benefits from extensive clinical experience demonstrating its safety and efficacy in treating vascular malformations. However, its drawbacks include temporary loss of visibility during longer injections, significant imaging artifacts on cross-sectional imaging due to its tantalum content, and longer preparation time. Squid, a newer copolymer similar to Onyx, offers improved visibility and extra-low viscosity versions but produces comparable imaging artifacts. Coils and polyvinyl alcohol (PVA) particles may be used as adjuncts but are rarely employed alone for high-flow AVFs due to recanalization risk.

PHIL offers several potential advantages over these established agents [10, 12]. Unlike Onyx and Squid, PHIL has iodine covalently bonded to the copolymer, eliminating the need for tantalum powder and resulting in significantly fewer artifacts on post-procedural CT. It is ready to use without shaking, requires shorter preparation time, and demonstrates more stable visibility during injection. Experimental studies suggest that PHIL achieves comparable embolization success to Onyx but with lower volumes of embolic agent required. Clinical series have reported complete occlusion rates of 77–85% for cranial vascular malformations, with safety profiles comparable to Onyx. The main limitation of PHIL, however, is the relative paucity of long-term efficacy and safety data compared to the extensive experience accumulated with Onyx and cyanoacrylates over decades.

The choice of embolic agent must therefore be individualized based on the specific angioarchitecture of the fistula, operator experience, and desired balance between complete occlusion and complication risk. In the present case, PHIL was selected due to its favorable penetration characteristics in high-flow fistulas, predictable precipitation, and the advantage of minimal post-procedural imaging artifacts for follow-up surveillance. The application of PHIL in this context represents a promising advancement in endovascular therapy, offering an effective, minimally invasive alternative to traditional embolic materials while acknowledging that longer-term data are needed to establish its comparative effectiveness.

The rarity of TMJ arthroscopy-related AVFs underscores the importance of preventive measures [2]. Surgeons should adopt meticulous techniques, including preoperative vessel palpation, precise instrument placement, and avoidance of excessive force during arthroscopic maneuvers. Additionally, the lateral puncture approach, has been advocated as a means to minimize inadvertent vascular trauma [8].

## Conclusion

While TMJ arthroscopy remains a safe and effective intervention for various TMJ disorders, the potential for vascular complications, including AVFs, should not be underestimated. Early recognition, prompt imaging evaluation, and appropriate therapeutic interventions are crucial in mitigating morbidity associated with these rare but serious complications. PHIL embolization, a newer endovascular technique utilizing a non-adhesive liquid embolic agent, has shown promise in treating AVFs by effectively penetrating and occluding abnormal vascular channels while minimizing the risk of non-target embolization.

## Supplementary Information

Below is the link to the electronic supplementary material.

Supplementary Material 1

Supplementary Material 2

## Data Availability

The data sets generated during and/or analysed during the current study are available from the corresponding author on reasonable request.
